# Bevacizumab improves survival in metastatic colorectal cancer patients with primary tumor resection: A meta-analysis

**DOI:** 10.1038/s41598-019-56528-2

**Published:** 2019-12-30

**Authors:** Dedong Cao, Yongfa Zheng, Huilin Xu, Wei Ge, Ximing Xu

**Affiliations:** 1 Department of Oncology, RenMin Hospital of Wuhan University, Jiefang Road #238 Wuchang District, Wuhan, 430000 China; 2 Department of Oncology, The Fifth hospital of Wuhan, Xianzheng Street #122 Hanyang District, Wuhan, 430000 China

**Keywords:** Targeted therapies, Colorectal cancer, Surgical oncology

## Abstract

It is not well determined whether primary tumor resection is associated with better outcomes in metastatic colorectal cancer (mCRC) patients treated with bevacizumab. In this meta-analysis, we aimed to assess the prognostic role of primary tumor resection in mCRC treated with bevacizumab. Electronic databases including the Cochrane library, Embase, and Pubmed were searched until April 2018. Clinical studies assessing the influence of primary tumor resection on the efficacy of bevacizumab in patients with mCRC were identified. The primary endpoint was overall survival (OS), and the secondary endpoint was progression-free survival (PFS). Seven studies including 2760 mCRC patients were finally included. The results of the meta-analysis were in favor of bevacizumab to patients with resected primary tumor in terms of OS (HR = 0.50, 95%CI: 0.39–0.64; *p* < 0.01), and PFS (HR = 0.65, 95%CI: 0.51–0.81; *p* < 0.01). Administration of bevacizumab in mCRC patients with resected primary tumor had a better OS (HR = 0.65, 95%CI: 0.56–0.74; *p* < 0.01), when compared to chemotherapy(CT). Adding bevacizumab to mCRC patients without resection of primary tumor also had a better OS (HR = 0.78, 95%CI: 0.65–0.94; *p* < 0.01) and PFS (HR = 0.71, 95%CI: 0.57–0.88; *p* < 0.01) compared to chemotherapy alone. In conclusion, mCRC patients with resected primary tumor have better survival than those without surgery of primary tumor when treated with bevacizumab. Primary tumor resection status should be taken into consideration when using bevacizumab in mCRC.

## Introduction

Colorectal cancer (CRC) is one of the most common tumors, and 20%~25% of these patients are diagnosed as stage IV disease^1^. CRC patients with unresectable metastases have a limited median survival around 5 months if only treated with best supportive care^2^. Due to effective treatment strategies, the survival of mCRC has been extensively improved. Bevacizumab, one of the molecular targeted drugs, brings survival advantage in metastatic CRC (mCRC) as proved by recent pieces of evidence^3,4^. However, not all the mCRC patients could get clinical benefits from bevacizumab. How to predict the efficacy of bevacizumab in mCRC is still under investigating^5^.

Previously, several studies and reviews^6–8^ have shown that primary tumor resection is associated with better outcomes in mCRC patients after treatment of chemotherapy or radiotherapy. Ishihara *et al*.^6^ reported that primary tumor resection significantly improved cancer-specific survival (HR = 0.46, p < 0.01). The study of Tong^8^ also concluded that surgery of primary cancer might improve the survival of metastatic rectal cancer patients. However, whether primary tumor resection also has the same role in predicting the efficacy of bevacizumab, it has not been well investigated.

In recent years, there are accumulating clinical studies^9–15^ that assessing the efficacy of bevacizumab is influenced by primary tumor resection or not in mCRC. These studies suggested that surgery of primary tumor was associated with better survival when managed with bevacizumab. Although positive results emerged, an agreement about the impact of primary tumor resection on bevacizumab has not yet been reached^3,16,17^. These studies are not capable of providing encouraging evidence for guiding use of bevacizumab or predicting efficacy of bevacizumab based on primary tumor resection in mCRC.

Thus, we identified clinical trials assessing the impact of primary tumor resection on the efficacy of bevacizumab in mCRC patients and performed a meta-analysis by using HRs of resection versus no resection for survival in mCRC patients after bevacizumab treatment. With this purpose, we expected to establish an evidence-based relationship between primary tumor resection and efficacy of bevacizumab.

## Methods

### Search strategy

The Preferred Reporting Items for Systematic Reviews and Meta-Analyses guidelines(PRISMA)^18^ was applied during the process of this meta-analysis. There was no registration information for this study. Electronic databases including the Cochrane library, Embase, and Pubmed were searched to identify clinical trials assessing the impact of resection versus no resection of primary tumor on the survival of mCRC patients with a deadline of April 2018. The search terms of (“colonic neoplasm”, or “tumor, colorectal”, or “carcinoma, colorectal”, or “colorectal cancer”), (“survival”, or “overall survival”, or “progression-free survival”, or “OS”, or “PFS”), (“bevacizumab” or “ Avastin”, or “anti-VEGF humanized monoclonal antibody”), and (“primary tumor resection” or “primary tumor surgery” or “resected primary tumor” or “original tumor resection” or “original tumor surgery”) were used in different combinations during the search. The “similar articles” indicated by Pubmed was also reviewed to achieve a maximum inclusion. There was no language limitation in this study. Conference abstracts were also included if sufficient data was provided.

### Study selection, inclusion and exclusion criteria

The primary endpoint was overall survival (OS), while the secondary was progression-free survival (PFS). Clinical trials evaluating the relationship between primary tumor resection and benefits of bevacizumab on survival in mCRC patients were included. The inclusion criteria were: (1) clinical studies that included mCRC patients, either randomized controlled trials or retrospective studies with sufficient baseline and endpoint information; (2) mCRC patients treated with bevacizumab in combination with or without traditional chemotherapy; (3) clear definition of OS and PFS; (4)Comparing impact of primary tumor resection versus no resection on OS and/or PFS in mCRC treated with bevacizumab; (5) sufficient data for extracting hazard ratio(HR) and its 95% confidence interval(CI) in term of OS and PFS directly or indirectly(survival curve provided). Reviews, animal studies, treatment guidelines, case reports, letters, comments, and meta-analyses were excluded. Studies without sufficient data to calculate the HRs and associated CIs were also discarded.

### Data extraction and quality assessment

The work of data extraction was conducted by two reviewers (Dedong Cao, Huilin Xu), independently. A third reviewer was invited if there were any disagreements during the process of data extraction. Baseline characteristics such as first author, publication year, number of included patients, gender, age, region, treatment line and dose of bevacizumab, and chemotherapy were collected. Primary and secondary endpoints including OS and PFS were also extracted. The reported methods^19,20^ were applied to obtain HRs and related CIs if they were not directly presented in the included studies. The first author and publication year were used to identify included studies.

The Newcastle Ottawa Scale (NOS) that recommended by the Cochrane non-randomized studies methods working group was applied to evaluate the quality of included studies^21^. In brief, NOS had three broad perspectives and they were selection, comparability, and outcome. There were four, one and three numbered items to assess the quality of retrospective studies, respectively. A study can be awarded a maximum of one star for each numbered item within the Selection and Outcome categories. A maximum of two stars can be given for Comparability^22^. In this study, we defined a high-quality study if it met five or more NOS criteria item. This work was finished by Dedong Cao and Huilin Xu, independently. Another researcher was involved if a discrepancy about quality evaluation of a certain study emerged.

### Statistical analysis

To perform the statistical analysis, we used the RevMan 5.3 and STATA 12 software from the Cochrane Collaboration. The endpoints were OS and PFS. The HRs and relevant 95% CIs of resection versus no resection of primary tumor for assessing the effect of resection on OS and PFS in bevacizumab treated mCRC were extracted from full-text or survival curves. The logarithms of HRs (logHRs) along with the standard error (SE) were calculated to conduct the combined analysis. Before performing the synthesized analysis, I^2^ statistics by Higgins^23^ was used to detect the potential heterogeneity between included studies. The percentage of variation across studies that caused by heterogeneity rather than chance was described by I² statistic^23^. The significance of heterogeneity indicated by I^2^ statistics was classified into three degrees as previously described^23^. In brief, it was considered as low, moderate, or high heterogeneity if the I^2^ < 50%, 50% < I^2^ ≤ 75%, and I^2^ > 75%, respectively. For moderate and high risk of heterogeneity, the random effect model (Mantel-Haenszl) was applied. Otherwise, the fixed effect model (Mantel-Haenszl) was used. For analysis with moderate and high risk of heterogeneity, the subgroup analysis and sensitivity analysis were introduced to find the possible sources of differences. The items used to perform the subgroup analysis were region, treatment line of bevacizumab, and dose of bevacizumab. If the combined HR < 1, it indicated a better efficacy of bevacizumab for the primary tumor resected mCRC patients. The funnel plot was used to check the risk of publication bias. All statistical tests were two-sided, and a statistical significance was achieved if the p < 0.05.

### Statement of significance

Although bevacizumab prolonged the survival of colorectal cancer (CRC) patients, it is still difficult to predict whether the survival of metastatic patients can be significantly improved by bevacizumab. Recently, there is a debate about the impact of primary tumor resection (PTR) on efficacy of bevacizumab in metastatic CRC (mCRC). While some researchers suggested a positive role of PTR on predicting survival advantage of bevacizumab, other investigators found negative results. Therefore, we used meta-analysis to determine the effects of PTR versus no PTR in mCRC patients after treatment with bevacizumab, aimed to establish a prognostic role of PTR in predicting efficacy of bevacizumab, and gave reliable recommendation on surgical treatment of primary tumor if possible. Our findings suggested that mCRC patients with PTR had a significantly better survival after bevicazumab treatment. These findings indicated PTR can be recommended as a prognostic factor of good efficacy of bevacizumab in mCRC patients.

## Results

### Search results

**Figure 1:**
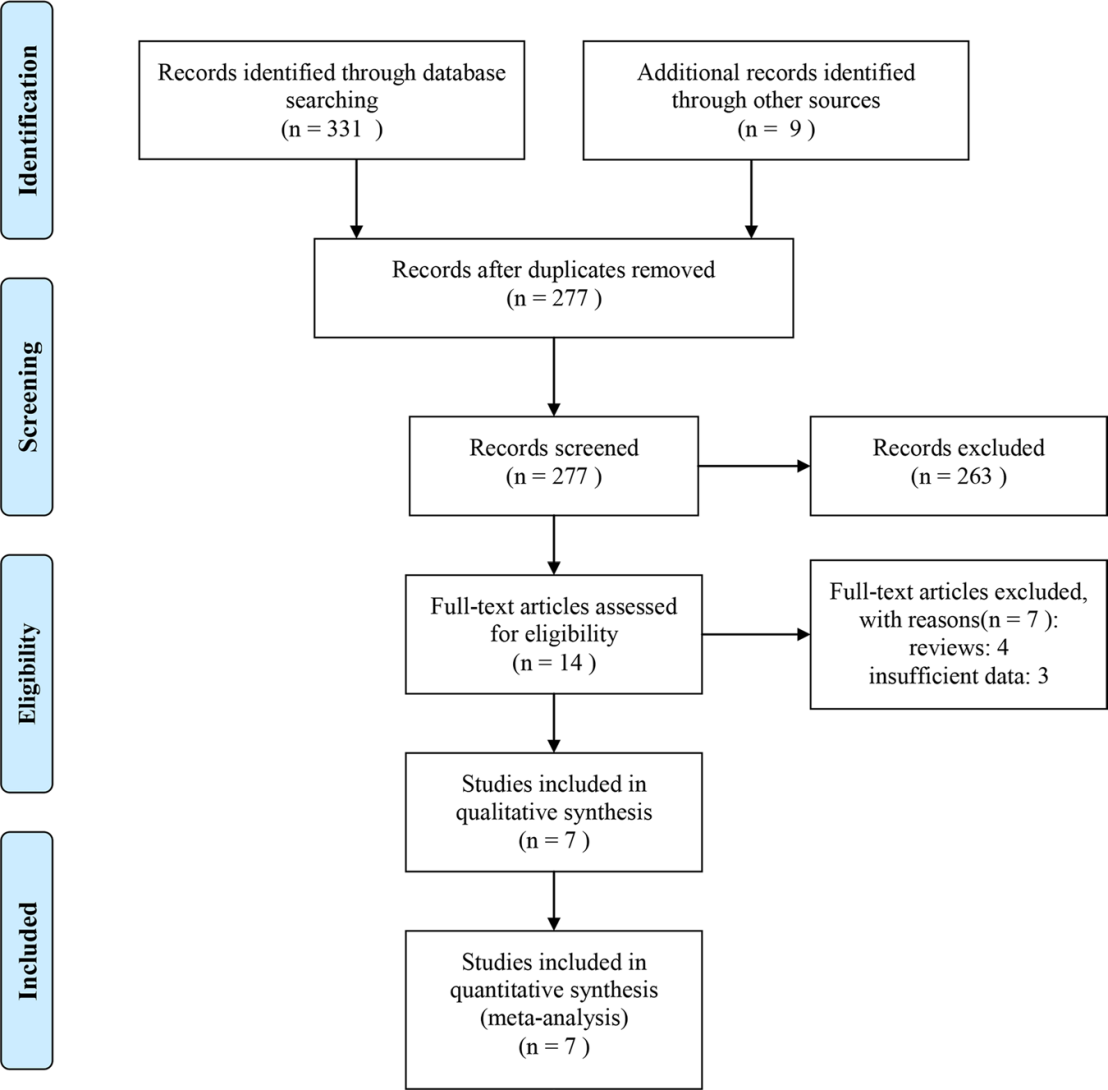
Flow diagram of searching for eligible studies.

After a comprehensive search, a number of 340 studies were retrieved. 63 of them were duplicates and discarded. 277 studies were screened with title and abstract, and 263 of them were excluded as they were animal studies, reviews, case-report, guidelines, commentary, and meeting abstracts with limited data. The left 14 studies were further reviewed by full-text, and half of them were excluded as they were lack of sufficient data and reviews. Finally, seven clinical studies^2,3,11,14,16,17,24^ were regarded as eligible and included for the final quantitative synthesis. The detailed study selection process is presented in Fig. 1.

### Baseline characteristics of included studies

Among seven included studies, five^3,11,14,16,24^ of them were retrospective trials and two^2,17^ were prospective studies published from 2011 to 2016. The median age ranged from 52 to 66. The most used chemotherapies were fluorouracil, oxaliplatin-based or irinotecan-based regimen (IFL, bolus 5FU+ irinotecan; FOLFIRI, infusional 5FU + irinotecan; XELOX,capecitabine+ oxaliplatin; FOLFOX, 5FU + oxaliplatin + leucovorin). The dose of bevacizumab ranged from 5 mg/kg to 7.5 mg/kg, and the treatment line ranged from the first line to third or more line. Some the primary tumor site data were not available(NA) from some of the included studies. Adverse events(AEs) were also reported in some of the studies. The individual OS reported by each included study ranged from 20.7 to 29.8 months in the resection group, whereas they were from 13.4 to 20 months in the no resection group (Table 1). The PFS was between 9 and 10.8 months in the resection group, while they were between 7 and 9 months in no resection group. Few of the included trials reported HR directly, while HRs were calculated from survival curves from other studies^2,3,11,14,16,17^, indirectly. The method was mentioned in the statistical analysis section. The baseline characteristics of the included studies are presented in Table 1.

**Table 1:** Baseline characteristics of mCRC patients with primary tumor resection(PTR) versus intact primary tumor(IPT).

Authors	Year	N	Participants		Age(year)		Sex		Treatment		Treatment line	Primary tumor		Outcomes	OS(months)		PF S(months)	
			PTR	IPT	PTR	IPT	male	female	CT	Bevacizumab (Bev)	Bev	Colon	Rectum		PTR	IPT	PTR	IPT
Sabine	2011	448	289	159	62 (35–80)	60 (31–78)	260	188	XELOX	7.5 mg/kg every 3 weeks	First	199	84	OS, PFS, AEs	20.7	13.4	10.5	7.8
Bulent	2013	99	53	46	55 (28–73)	52 (23–74)	56	43	FOLFIRI/XELOX/IFL	Median cumulative bevacizumab doses were similar between the groups: non-surgery group 4400 mg (range, 1200–12 800) vs. surgery group 4800 mg (range, 1600–9200)	Multiple line	65	34	OS, PFS, AEs	23	17	9	7
Francois	2014	409	193	41	66 (24–90)	65 (35–86)	NA	NA	FOLFIRI/XELOX/IFL	Either 5 mg/kg every 2 weeks or 7.5 mg/kg every 3 weeks	Multiple line	184	48	OS	27	20	NA	NA
Kodaz	2015	93	46	47	60 (30–82)	61 (27–84)	52	41	FOLFIRI/Folfox based	Used as suggested dose	Multiple line	NA	NA	OS,PFS	25	16	9	9
Lee	2016	1204	357	200	66 (25–92)	62 (27–89)	220	337	FOLFIRI/Oxaliplatin-based/Irinotecan-based	7.5 mg/kg every 3 weeks	Multiple line	381	148	OS, PFS, AEs	24.4	20	10.8	8.5
Mathilde	2016	316	164	52	65 (31–83)	63 (22–87)	138	78	Oxaliplatin-based/Irinotecan-based	Either 5 mg/kg every 2 weeks or 7.5 mg/kg every 3 weeks	Multiple line	158	54	OS, PFS, AEs	29.8	18.2	9.7	8.1
Wang	2016	191	118	73	57 (22–77)	58 (22–73)	108	83	FOLFIRI/XELOX/FOLFOX	Either 5 mg/kg every 2 weeks or 7.5 mg/kg every 3 weeks	First	115	76	OS, PFS, AEs	22.5	17.8	10	7.8

The quality assessment results of the included studies are summarized in Table 2. All of the eligible studies were regarded as moderate or high quality as they met most of the criteria of the NOS.

**Table 2:** Quality assessment of included studies by NOS.

First author	Year	Selection	Comparability	Outcome
Sabine	2011	⋆⋆⋆	⋆⋆	⋆⋆⋆
Bulent	2013	⋆⋆⋆	⋆⋆	⋆⋆⋆
Francois	2014	⋆⋆	⋆⋆	⋆⋆⋆
Kodaz	2015	⋆⋆⋆	⋆⋆	⋆⋆⋆
Lee	2016	⋆⋆⋆	⋆⋆	⋆⋆⋆
Mathilde	2016	⋆⋆⋆	⋆⋆	⋆⋆⋆
Wang	2016	⋆⋆⋆	⋆	⋆⋆

Note: Retrospective studies were assessed by NOS method. A study can be awarded a maximum of one star for each numbered item within the Selection and Outcome categories. A maximum of two stars can be given for Comparability, according to the instruction of NOS.

### Results of meta-analysis

#### OS

**Figure 2:**
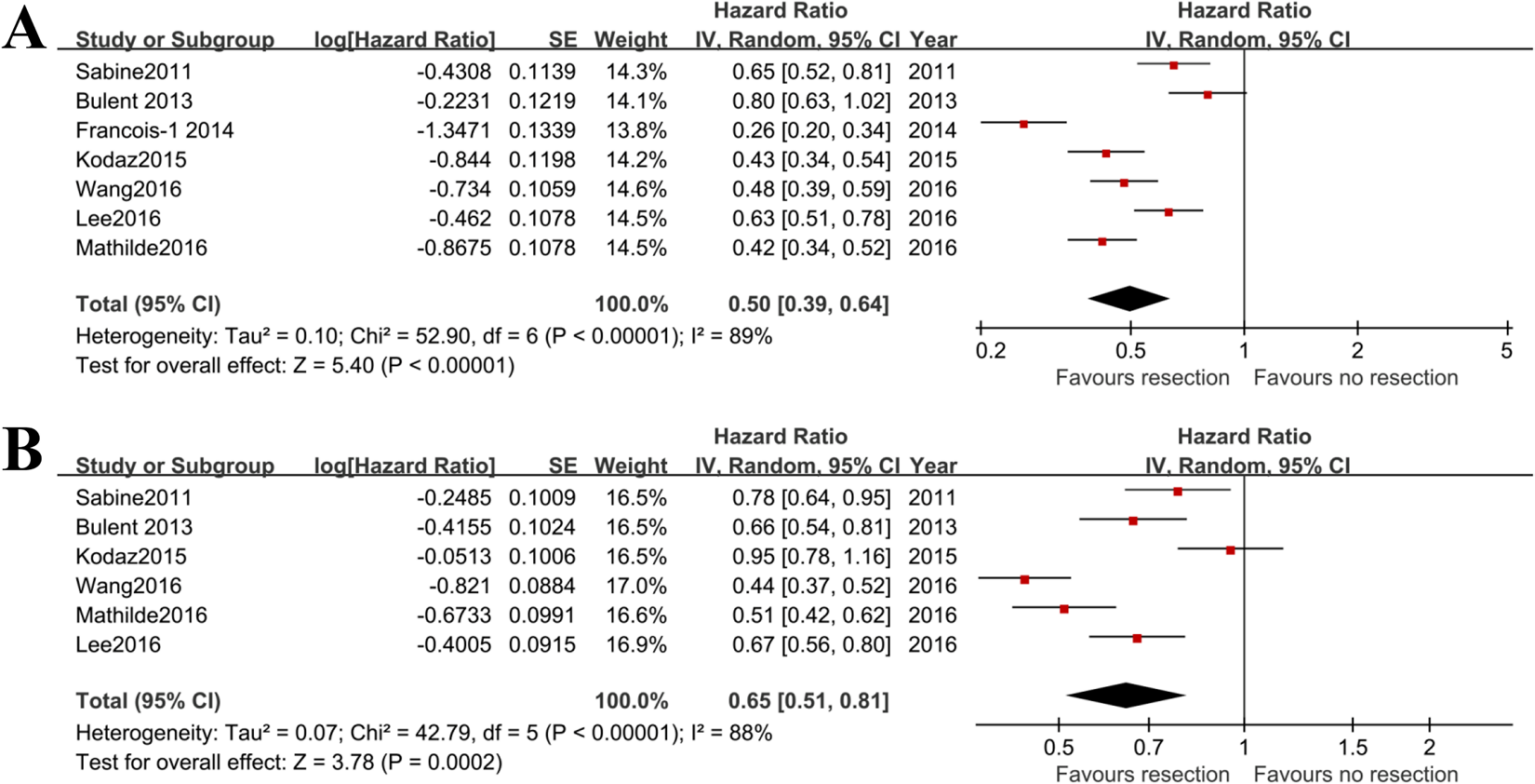
Pooled overall survival and progression free survival outcomes in mCRC patients with primary tumor resection versus no resection after treatment of bevacizumab. (**A**) combined HR of resection (n = 1220) versus no resection (n = 618) of primary tumor for assessing effect of bevacizumab on OS; (**B**) combined HR of resection (n = 1027) versus no resection (n = 577) of primary tumor for assessing effect of bevacizumab on PFS.

To evaluate the role of primary tumor resection on OS in mCRC patients who were treated with bevacizumab, the HRs of resection versus no resection were extracted from all of the included studies^2,3,11,14,16,17,24^. As indicated by the heterogeneity test (I^2^ = 89%), the random effect model was used. There were 1220 patients underwent primary tumor resection and 618 cases without resection. The included patients were all treated with bevacizumab. The results of meta-analysis showed that the risk of mortality in primary tumor resected mCRC patients was significantly reduced after treatment of bevacizumab (HR = 0.50; 95%CI: 0.39, 0.64; p < 0.00001), compared to those without primary tumor resection (Fig. 2A).

As there was significant heterogeneity, the subgroup analysis was introduced based on the factors of region (Supplemental Fig. 1A), high-dose (7.5 mg/kg every three-week) of bevacizumab (Supplemental Fig. 1B), and treatment line of bevacizumab (Supplemental Fig. 1C). The dose and treatment line of bevacizumab varied between included studies and high-dose subgroup of OS had a low heterogeneity (I^2^ = 0%). The sensitivity analysis revealed that the combined HR for OS was not significantly changed after exclusion of each included studies (Supplemental Fig. 2).

**Figure 3:**
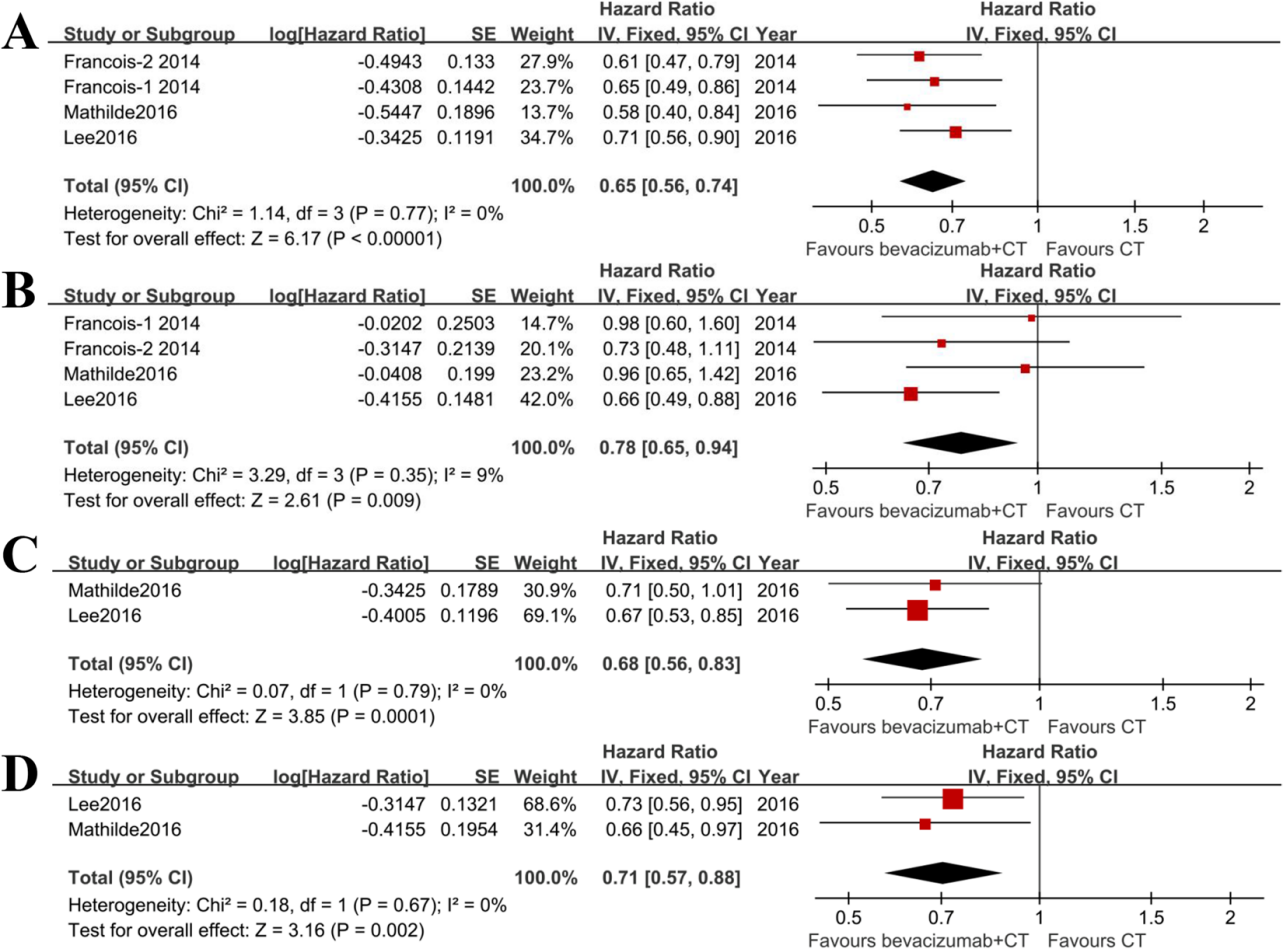
Combined HR to evaluate benefits of adding bevacizumab to chemotherapy versus chemotherapy alone in mCRC patients with or without primary tumor resection. (**A**) OS of bevacizumab plus chemotherapy versus chemotherapy alone in primary tumor resected patients (n = 847); (**B**) OS of bevacizumab plus chemotherapy versus chemotherapy alone in patients without resection of primary tumor (n = 424); (**C**) PFS of bevacizumab plus chemotherapy versus chemotherapy alone in primary tumor resected patients (n = 521); (**D**) PFS of bevacizumab plus chemotherapy versus chemotherapy alone in patients without resection of primary tumor (n = 252).

Next, we extracted the HRs of bevacizumab + chemotherapy versus chemotherapy alone for OS in primary tumor resected mCRC (Fig. 3A) and no resection (Fig. 3B) patients. Three studies^11,14,17^ provided specific data of interest. In primary tumor resected population, the administration of bevacizumab was associated with significantly improved OS when compared with chemotherapy (I^2^ = 0%, HR = 0.65; 95%CI: 0.56, 0.74; p < 0.00001). In patients with intact primary tumor, most of the included studies showed there were no significant benefits on OS when using bevacizumab (p > 0.05). However, the pooled OS was significantly increased after adding bevacizumab to chemotherapy (I^2^ = 9%, HR = 0.78; 95%CI: 0.65, 0.94; p = 0.009). The result from Lee *et al*.^17^ contributed most to this improvement in OS (Supplemental Table 1).

#### PFS

A meta-analysis of PFS between resection versus no resection of primary tumor in bevacizumab treated mCRC was performed. Six studies^2,3,11,16,17,24^ reported relevant HR or survival curve. The random effect model was used as there was a high risk of heterogeneity (I^2^ = 88%). There were 1027 patients had the primary tumor resection and 577 patients with no resection of the primary tumor. Bevacizumab was administrated to all the included patients. Results showed that the risk of progression was decreased by 0.35(HR = 0.65; 95%CI: 0.51, 0.81; p = 0.0002) in resected patients after receiving bevacizumab, compared to no resection of primary tumor (Fig. 2B).

To assess the influence of bevacizumab on PFS in primary tumor resected and no resected patients, the data of interest was extracted from two studies^11,17^. The fixed effect model was used for these two meta-analyses (Fig. 3C,D). Adding bevacizumab to chemotherapy could statistically improve PFS in mCRC patients either with (HR = 0.68; 95%CI: 0.56, 0.83; p = 0.0001) or without (HR = 0.71; 95%CI: 0.57, 0.88; p = 0.002) primary tumor resection (Supplemental Table 1).

#### Publication bias assessment

To assess the potential publication bias, the funnel plot along with the Begg’s test and Egger’s test were used. As illustrated by Supplemental Fig. 2, the funnel chart was symmetrical. The Begg’s test (p = 1.0) and Egger’s test (p = 0.416) suggested there was no significant publication bias with regards to OS.

## Discussion

Whether primary tumor resection could affect the survival outcome of mCRC patients treated with bevacizumab, it was still in debate. To address this concern, we performed this meta-analysis by including individual data from seven clinical studies that assessing the impact of primary tumor resection on survival benefit of bevacizumab in mCRC patients. The results of meta-analysis showed that mCRC patients with resected primary tumor had a significantly better OS and PFS compared to those without resection of primary tumor. In addition, when compared with chemotherapy alone, the addition of bevacizumab to chemotherapy also significantly improved OS and PFS in primary tumor resected mCRC patients. The combined effect of bevacizumab on OS in mCRC patients without resected primary tumor was positive. However, this evidence was not so convincing. These findings suggested that primary tumor resection was associated with significantly better efficacy of bevacizumab in terms of OS and PFS, and it could be used as a prognostic factor of bevacizumab in mCRC patients (Supplemental Table 1). To our knowledge, this is the first meta-analysis evidence to show the role of primary tumor resection on survival of mCRC patients treated with bevacizumab.

The present study found that the efficacy of bevacizumab was influenced by the resection status of primary tumor in mCRC patients. Patients with resected primary tumor could benefit most from bevacizumab. As shown above, after treating with bevacizumab, the individual OS reported in primary tumor resected patients ranged from 20.7 months to 29.8 months, and they were from 13.4 months to 20 months in no resection patients. The combined median OS was 24.6 months for resection and 17.5 months for no resection, with a difference of 7.1 months. The difference of median PFS was 1.8 months in term of resection versus no resection. The pooled results demonstrated a longer OS after bevacizumab treatment in resection group than that of the no resection group. In addition, in patients with primary tumor resection, the OS was also significantly improved after adding bevacizumab to chemotherapy versus chemotherapy alone. The subgroup analysis suggested that the impact of primary tumor resection on OS was not statistically changed by region, treatment line and dose of bevacizumab, suggesting primary tumor resection was a reliable indicator for better outcomes of bevacizumab.

In most current guidelines, the role of primary tumor resection in mCRC patients has not been well established^25,26^. For stage IV CRC, multiple factors should be taken into consideration before making a treatment decision. The guidelines of EURECCA and ESMO do not suggest resecting primary tumor in asymptomatic mCRC patients that beyond resection possibilities^25^. These recommendations are established based on the evidence provided by Cirocchi *et al*.^27^. That meta-analysis found that resection of primary tumor in asymptomatic mCRC who were treated with chemo/radiotherapy was not associated with a consistent improvement in OS^27^. However, recent meta-analyses^26,28–31^ demonstrate that primary tumor resection may provide a survival benefit. These studies assessed the role of primary tumor resection on efficacy of chemotherapy or radiotherapy, but not bevacizumab. Therefore, we perform this meta-analysis to address whether the efficacy of bevacizumab is also influenced by primary tumor resection. A positive link between resection of primary tumor and effects of bevacizumab on survival was confirmed by our findings. Several clinical indications are concluded from the present meta-analysis. Clinically, the primary tumor should be resected if possible. And the resection status can be used as a prognostic factor to predict the beneficial effects of bevacizumab containing regimen. It is also encouraged to adding bevacizumab to chemotherapy in original tumor resected mCRC patients.

There are several limitations in our meta-analysis. First, high risk of heterogeneity existed among the included trials. The differences of baseline characteristics, chemotherapy regimen, treatment line and dose of bevacizumab, sites of primary tumor, numbers and sites of metastasis may contribute to the significant heterogeneity. Second, though all of the studies were considered as moderate or high quality, only a few of them reported HRs of primary and secondary endpoints, directly. Data from the survival curve had to be extracted to calculate the HR, indirectly. In this condition, the strength of our findings is hampered due to the less reliable HR. Even with these shortcomings, our results still provide objective evidence about the relationship between primary tumor resection and efficacy of bevacizumab.

In conclusion, this meta-analysis suggests that mCRC patients with resected primary tumor have better survival when managed with bevacizumab. Randomized controlled trials are needed to confirm our findings.

## Supplementary information

Supplemental Table 1

Supplemental Figures
