# Supplementary material for: Bevacizumab improves survival in metastatic colorectal cancer patients with primary tumor resection: A meta-analysis
Source: Sci Rep. 2019 Dec 30;9:20326. doi: 10.1038/s41598-019-56528-2 (PMC6937309; doi:10.1038/s41598-019-56528-2)
Supplement: Supplementary file 1 — Supplemental Table 1 [file 41598_2019_56528_MOESM1_ESM.docx]

**Bevacizumab improves survival in metastatic colorectal cancer patients with primary tumor resection: a meta-analysis**

**Running title: Primary tumor resection influences the efficacy of bevacizumab**

**Dedong Cao^1*#^, Yongfa Zheng^1#^, Huilin Xu^2^, Wei Ge^1^, Ximing Xu^1*^**

^1^, Department of Oncology, RenMin Hospital of Wuhan University, Jiefang Road #238 Wuchang District, Wuhan, 430000, China.

^2^, Department of Oncology, The Fifth hospital of Wuhan, Xianzheng Street #122 Hanyang District, Wuhan, 430000, China.

**# These two authors contributed equally.**

*** Corresponding authors:**

Dedong Cao, Phone: 86 15927564963, E-mail address: caodedong123@163.com

Ximing Xu, Phone: 86 13707120651, E-mail address: doctorxu120@aliyun.com

**Supplemental Table 1 Summarized results of impact of primary tumor resection on survival in mCRC patients**

| **Outcome or Subgroup** | **Studies** | **Statistical Method** | **Effect Estimate** |
| --- | --- | --- | --- |
| **1 resection vs no resection in mCRC patients treated with bevacizumab** | | | |
| 1.1 OS | 7 | Hazard Ratio (IV, Random, 95% CI) | 0.50 [0.39, 0.64] |
| 1.2 PFS | 6 | Hazard Ratio (IV, Random, 95% CI) | 0.65 [0.51, 0.81] |
| 1.3 OS region subgroup analysis |  |  |  |
| 1.3.1 Asia | 3 | Hazard Ratio (IV, Random, 95% CI) | 0.55 [0.38, 0.79] |
| 1.3.2 Europe | 3 | Hazard Ratio (IV, Random, 95% CI) | 0.42 [0.25, 0.68] |
| 1.3.3 Australia | 1 | Hazard Ratio (IV, Random, 95% CI) | 0.63 [0.51, 0.78] |
| 1.4 OS dose subgroup analysis |  |  |  |
| 1.4.1 high dose | 2 | Hazard Ratio (IV, Random, 95% CI) | 0.64 [0.55, 0.75] |
| 1.4.2 multiple doses | 5 | Hazard Ratio (IV, Random, 95% CI) | 0.45 [0.32, 0.62] |
| 1.5 OS treatment line subgroup analysis |  |  |  |
| 1.5.1 First line | 2 | Hazard Ratio (IV, Random, 95% CI) | 0.56 [0.41, 0.75] |
| 1.5.2 Multiple line | 5 | Hazard Ratio (IV, Random, 95% CI) | 0.47 [0.33, 0.68] |
| **2 Bevacizumab+CT vs CT in resected primary tumor** | | | |
| 2.1 OS | 4 | Hazard Ratio (IV, Fixed, 95% CI) | 0.65 [0.56, 0.74] |
| 2.2 PFS | 2 | Hazard Ratio (IV, Fixed, 95% CI) | 0.68 [0.56, 0.83] |
| **3 Bevacizumab+CT vs CT in no resected primary tumor** | | | |
| 3.1 OS | 4 | Hazard Ratio (IV, Fixed, 95% CI) | 0.78 [0.65, 0.94] |
| 3.2 PFS | 2 | Hazard Ratio (IV, Fixed, 95% CI) | 0.71 [0.57, 0.88] |
