# Supplementary material for: Bevacizumab improves survival in metastatic colorectal cancer patients with primary tumor resection: A meta-analysis
Source: Sci Rep. 2019 Dec 30;9:20326. doi: 10.1038/s41598-019-56528-2 (PMC6937309; doi:10.1038/s41598-019-56528-2)
Supplement: Supplementary file 2 — Supplemental Figures [file 41598_2019_56528_MOESM2_ESM.docx]

**Bevacizumab improves survival in metastatic colorectal cancer patients with primary tumor resection: a meta-analysis**

**Running title: Primary tumor resection influences the efficacy of bevacizumab**

Dedong Cao^1*#^, Yongfa Zheng^1#^, Huilin Xu^2^, Wei Ge^1^, Ximing Xu^1*^

^1^, Department of Oncology, RenMin Hospital of Wuhan University, Jiefang Road #238 Wuchang District, Wuhan, 430000, China.

^2^, Department of Oncology, The Fifth hospital of Wuhan, Xianzheng Street #122 Hanyang District, Wuhan, 430000, China.

**# These two authors contributed equally.**

*** Corresponding authors:**

Dedong Cao, Phone: 86 15927564963, E-mail address: caodedong123@163.com

Ximing Xu, Phone: 86 13707120651, E-mail address: doctorxu120@aliyun.com


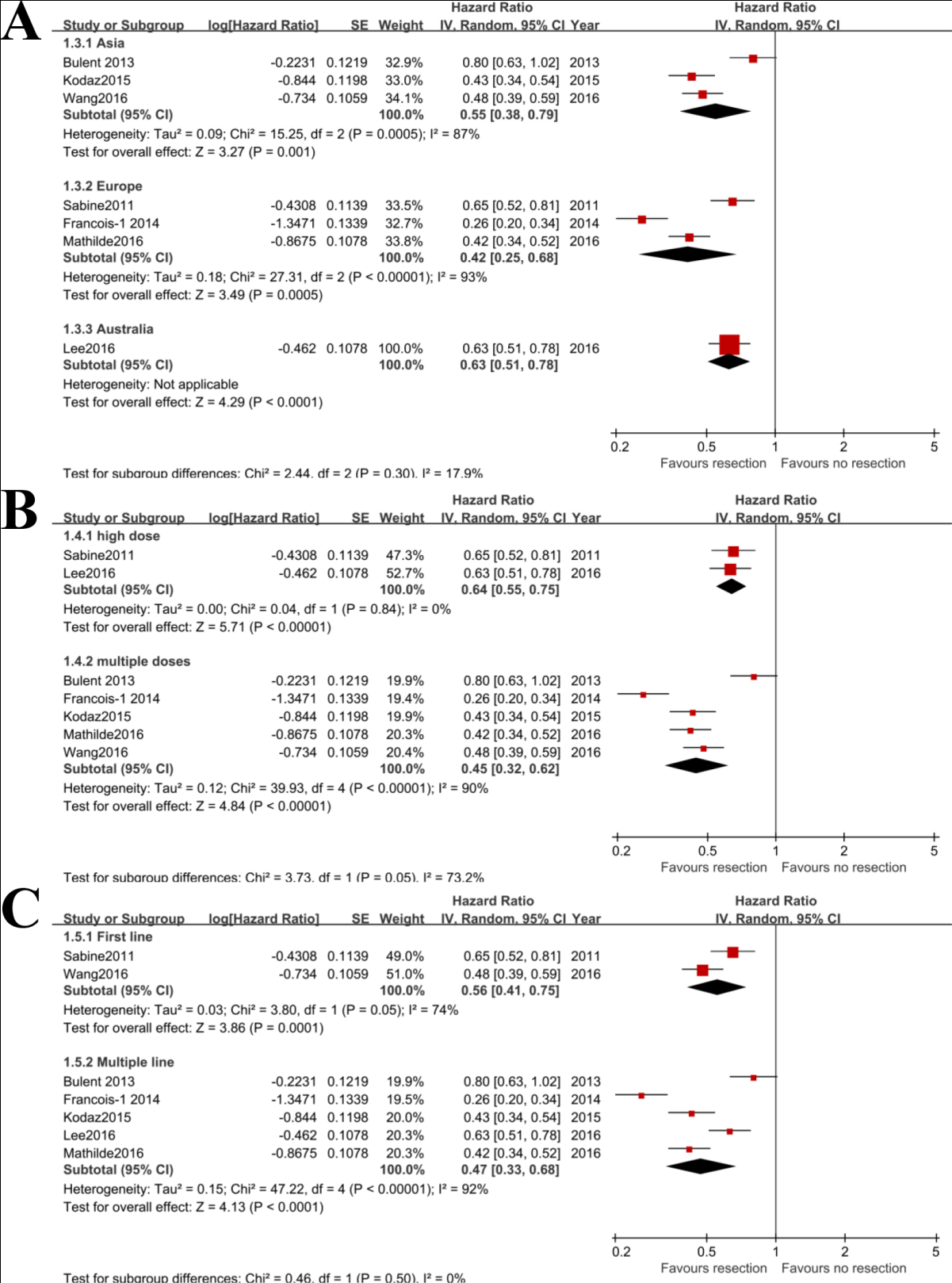


Supplemental Figure 1 Subgroup analyses of OS in terms of region, dose and treatment line of bevacizumab. A: region; B: dose of bevacizumab; C: treatment line of bevacizumab.


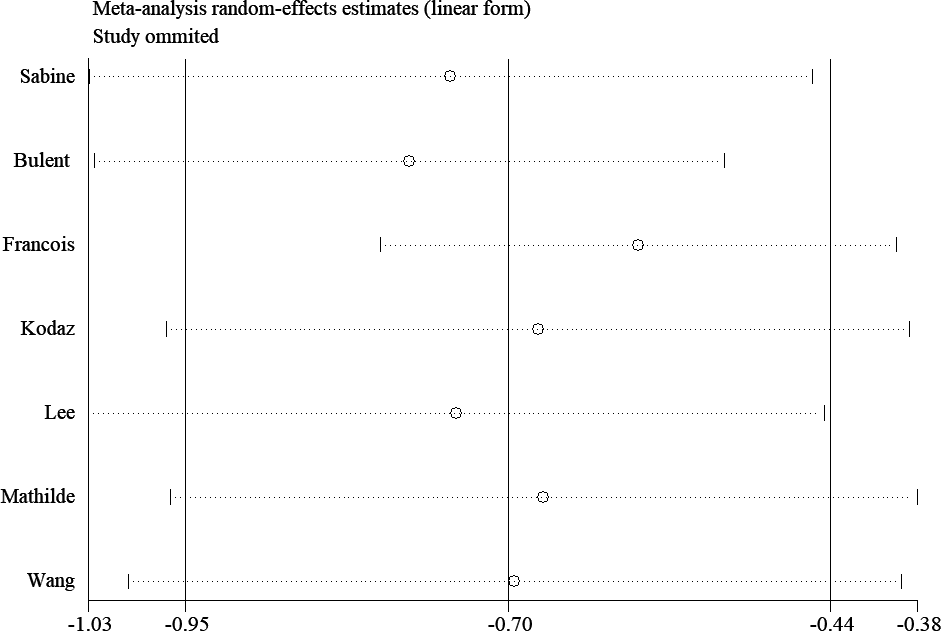


Supplemental Figure 2 Sensitivity analysis in term of OS


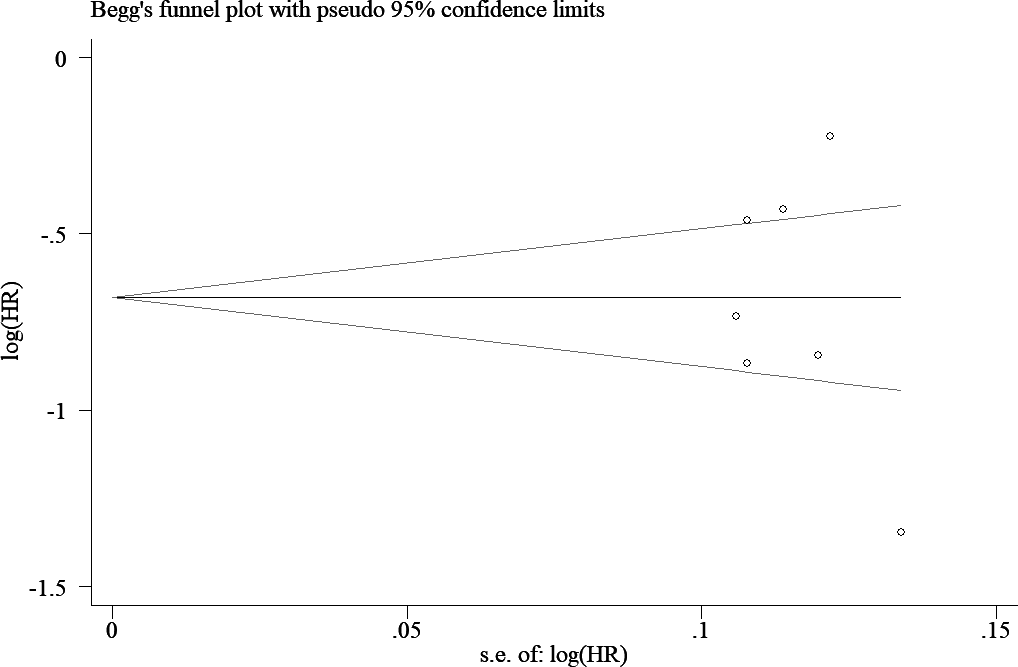


Supplemental Figure 3 Funnel plot for detecting publication bias
